# Usefulness of C-reactive protein/albumin ratio as a predictor of new-onset atrial fibrillation in SARS coronavirus-2

**DOI:** 10.2217/bmm-2020-0866

**Published:** 2021-08-18

**Authors:** Saban Kelesoglu, Yucel Yilmaz, Eyup Ozkan, Bekir Calapkorur, Zehra B Dursun, Aysegul Ulu-Kilic, Deniz Elcık

**Affiliations:** ^1^Department of Cardiology, Faculty of Medicine, Erciyes University, Kayseri, 38039, Turkey; ^2^Department of Cardiology, Kayseri City Hospital, Kayseri, 38080, Turkey; ^3^Department of Infectious Diseases, Kayseri City Hospital, Kayseri, 38080, Turkey; ^4^Department of Infectious Diseases, Faculty of Medicine, Erciyes University, Kayseri, 38039, Turkey

**Keywords:** C-reactive protein/albumin ratio, new-onset atrial fibrillation, SARS-CoV-2

## Abstract

**Aim:** To investigate whether C-reactive protein/albumin ratio (CAR) has an association with new onset atrial fibrillation (NOAF) in SARS-CoV-2. **Materials & methods:** This study included 782 patients with SARS-CoV-2 infection, who were hospitalized in Turkey. The end point of the study was an occurrence of NOAF. **Results:** NOAF was identified in 41 patients (5.2%). Subjects who developed NOAF had a higher CAR compared with those who did not develop NOAF (p < 0.001). In the multivariate logistic regression analysis the CAR (odds ratio = 2.879; 95% CI: 1.063–7.793; p = 0.037) was an independent predictor of NOAF. **Conclusion:** A high level of CAR in blood samples is associated with an increased risk of developing NOAF in SARS-CoV-2.

COVID-19 is caused by SARS-CoV-2 and is a new RNA virus that primarily presents with acute pneumonia and severe respiratory distress syndrome. Although SARS-CoV-2 primarily affects lungs, cardiovascular involvement has also been reported extensively [1].

Acute cardiovascular events that complicate the clinical course of SARS-CoV-2 may be one of the causes of poor survival. Arrhythmias are one of the most common cardiac complications during this illness [2]. The most common cardiac rhythm disorder in clinical practice, atrial fibrillation (AF), is a critical condition that is associated with hemodynamic disorders and thromboembolic events [3]. However, data on new onset AF (NOAF) in SARS-CoV-2 are lacking and the exact mechanisms leading to AF development are not fully understood. Besides known risk factors (such as age, hypertension [HT] and coronary artery disease), inflammation and inflammatory factors have also been shown to be associated with the development of AF [3–6].

The accumulated evidence has shown that besides the increase in C-reactive protein (CRP) level, the increase in new inflammatory markers such as the neutrophil/lymphocyte (L) ratio (NLR), platelet/L ratio (PLR) and CRP/albumin ratio (CAR) identified in recent years, is also associated with the development of NOAF [7–11].

The purpose of this study was to investigate NOAF predictors in patients hospitalized for SARS-CoV-2, who developed NOAF on follow-up.

## Materials & methods

This study was designed as a single-center prospectively study regarding confirmed SARS-CoV-2-infected patients, who were monitored and treated in our clinics and those in the intensive care units (ICUs) between 1 June 2020 and 20 September 2020. The study was carried out in an institute designated as a ‘SARS-CoV-2 Hospital’ by the Turkish Ministry of Health, to admit possible or confirmed cases of SARS-CoV-2. Only cases that were confirmed by molecular methods were included in the study. The study plan was approved by the institutional ethics committee and was conducted in accordance with the Helsinki declaration. Informed consent for inclusion into this study was obtained from all individual participants.

### Study population

Patients, older than 18 years of age, who received a SARS-CoV-2 infection diagnosis via PCR tests and had sinus rhythm at admission according to 12-lead ECG, were included in this study. Patients were excluded from the study if any of the following criteria applied: pre-existing permanent or persistent AF and patients with a previous history of AF attack, presence of malignancy, severe sepsis or immunosuppression, pregnancy, or breastfeeding. Severe renal or liver disease patients were also excluded from the study.

According to the ‘COVID-19 Diagnosis and Treatment Guide’, printed by the Turkish Ministry of Health, the clinical definition of patients was as follows: mild illness presents with features such as fever, muscle/joint pain, cough, sore throat and nasal congestion, with or without mild pneumonia, together with a respiratory rate <30/min and an O_2_ saturation above 90% while breathing room air. Severe illness is defined with widespread findings of pneumonia in computed tomography (CT). Patients, whose general condition deteriorated during their follow-up, required ICU. The routine criteria for ICU admission at our center were as follows (according to Ministry of Health guidelines): signs conclusive for severe respiratory failure including a SpO_2_ of ≤92% in ambient air, need for ≥6 l O_2_/min, need for noninvasive ventilation or invasive mechanical ventilation.

The presence of pneumonia was confirmed by CT imaging within 24 h of hospital admission for all patients. All SARS-CoV-2 patients in our study had signs of pneumonia on CT imaging. Examination of the radiological findings showed that the lesions tended to be located more in the peripheral and lower lobes. The most common radiological findings of the patients were bilateral ground glass, diffuse infiltration, consolidation and unilateral ground glass opacities. The standard SARS-CoV-2 infection treatment protocol recommended by Science Advisory Board of Turkish Ministry of Health, including oseltamivir phosphate 75 mg twice daily, hydroxychloroquine 200 mg twice daily and azithromycin 250 mg once daily (following a 500-mg loading dose), were administered to all patients. Demographic data, findings of the imaging studies and laboratory test results were retrieved from the institutional digital database.

Patients were divided into two groups as follows: patients, who developed AF during hospitalization (group 1) and patients, who did not develop AF during hospitalization (group 2). The primary outcomes of this study were the differences between these two groups in terms of demographic characteristics and laboratory measurements. The role of several risk factors in the development of NOAF was also evaluated via multivariate analysis.

### Rhythm follow-up

All patients had a baseline ECG at the time of hospitalization. Daily ECG was taken 12 h after the first drug dose and on the following days in all patients. A QT interval (QTc) exceeding 500 ms was accepted as the warning limit.

The aim of the study was the finding of an occurrence of any episode of NOAF within the period of hospitalization as documented by medical records, ECGs, rhythm strips and Holter monitors accordingly. All patients hospitalized in the ICU were followed up with a rhythm recording during their stay in the ICU. AF attacks lasting ≥30 s were considered NOAF, as defined in the European Cardiology guidelines [12]. The diagnosis of NOAF in other patients was determined according to ECG findings, evaluation of rhythm strips and medical records.

### Laboratory findings & echocardiographic imaging

Immediately after the diagnosis of SARS-CoV-2 infection, routine blood tests including serum troponin I, hemoglobin, white blood cells (WBCs) and platelets (PLT), were performed at the time of hospitalization, and results were retrieved from the institutional digital database. Control troponin I levels measured in patients, who developed NOAF, were also found in the hospital registry system and were considered normal if they were below the upper reference limit of the 99th percentile. NLR was found by dividing the number of neutrophils by the number of Ls. PLR was found by dividing the platelet count by the number of Ls. Similarly, CAR was obtained by dividing the CRP level by the albumin level.

Conventional echocardiography was performed with a Philips Epiq 7 ultrasound system (Philips, MA, USA). To reduce the risk of SARS-CoV-2 infection transmission, conventional echocardiography was applied only to patients with suspected myocardial damage. Echocardiographic images were obtained from the parasternal and apical views The Teichholz method was used for the calculation of left ventricular ejection fraction.

### Statistical analysis

Statistical analyses were performed using SPSS version 21.0 (SPSS, Inc., IL, USA) software for Windows. The distribution of quantitative variables was checked with the Shapiro–Wilk test. Descriptive data were given as mean ± standard deviation and median (interquartile range [IQR]), depending on normality of distribution. Median and IQR were given when the variable did not follow normal distribution. The independent sample *t*-test was used for the comparison of normally distributed quantitative variables and the Mann–Whitney *U*-test was used for the comparison of non-normally distributed quantitative variables. The comparison of the distributions of categorical variables was performed with chi-square tests. The effects of different variables on the development of NOAF were calculated with univariate analysis. Variables for which the unadjusted p-value was <0.05 were included in the model for potential risk factors. The final multivariate model included age, CRP, WBC, CAR, NLR and PLR. The cut-off level of CAR in predicting NOAF formation was determined by performing receiver operating characteristic (ROC) curve analysis. The p-values equal to or lower than 0.05 were considered to demonstrate statistical significance.

## Results

**Figure 1.**
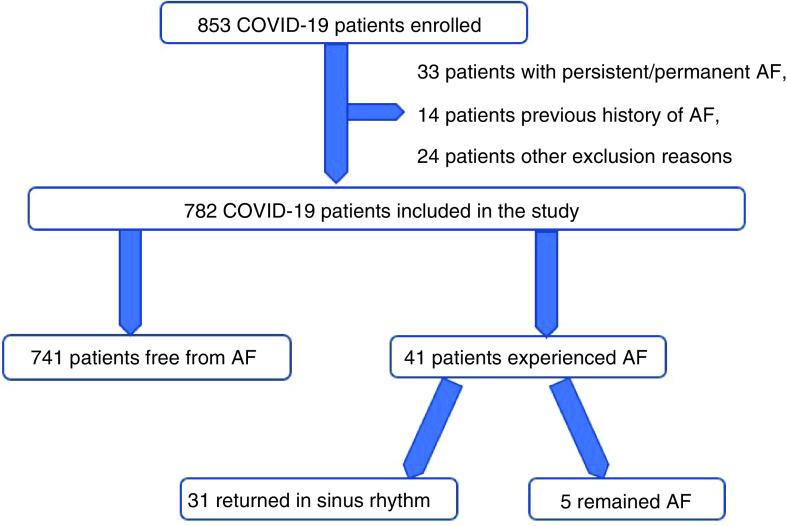
Patients’ study flow schema.

A total of 853 patients hospitalized for SARS-CoV-2 infection were screened for inclusion. Since the initial ECG of 33 patients was AF and 14 patients had a previous history of AF, 24 patients were not included because of the other exclusion reasons. The remaining 782 patients (91.6%) were included in the study (Figure 1). NOAF was identified in 41 patients (5.2%). In 37 patients, NOAF developed within the first 72 h of hospitalization and in the remaining four patients, NOAF developed within the first week of hospitalization. Among these 41 patients, 11 were followed up in the ICU. All the patients had hydroxychloroquine, azithromycin and favipiravir treatment. These patients had normal QTc (423 ms, average for all) intervals before treatment. After treatment, these values were QTc (467 ms, average for all). No patients needed to discontinue treatment due to QTc prolongation, and thus, there were no treatments applied for this condition.

Baseline demographic features of the study subjects are presented in Table 1. Patients, who developed AF, were older (median 59 years [IQR: 54–75] vs median 55 years [IQR: 46–66], p = 0.008) and had higher frequencies of HT and heart failure (HF) compared with patients without NOAF (46 vs 30%, p = 0.033; 29 vs 11%, p < 0.001, respectively). Other demographic characteristics were similar between both groups.

**Table 1.** Demographic and clinical characteristics of the study groups.

Characteristics	NOAF during hospitalization		
	Yes	No	p-value
Patients (n)	41 (5.2%)	741 (94.8%)	
Age (years)	59 (54–75)	55 (46–66)	**0.008**
Female gender (n, %)	21 (51%)	323 (44%)	0.338
Diabetes mellitus (n, %)	9 (22%)	121 (16%)	0.347
Hypertension (n, %)	19 (46%)	226 (30%)	**0.033**
Coronary artery disease (n, %)	6 (15%)	107 (14%)	0.973
Heart failure (n, %)	12 (29%)	79 (11%)	**<0.001**
Valvular heart disease (n%)	2 (4.8%)	26 (3.5%)	0.646
Smoking (n, %)	4 (10%)	85 (11%)	0.736
COPD/asthma (n, %)	6 (15%)	98 (13%)	0.796
Stroke/transient ischemic attack (n, %)	0	9 (1%)	0.478

Bold letters have a p-value less than 0.05 and are statistically significant.

COPD: Chronic obstructive pulmonary disease; NOAF: New onset atrial fibrillation.

The clinical parameters, laboratory and radiographic findings of the patients are presented in Table 2. Subjects, who developed NOAF during hospitalization, had higher CRP level, WBC count, NLR, PLR and CAR levels compared with those who did not develop NOAF (p < 0.001, p = 0.018, p = 0.010, p = 0.034, p < 0.001, respectively). Other blood parameters were similar between both groups.

**Table 2.** Laboratory findings and course of hospitalization and outcome of the study groups.

Characteristics	NOAF during hospitalization		
	Yes	No	p-value
Patients (n)	41 (5.2%)	741 (94.8%)	-
Glucose (mg/dl)	105 (90–117)	100 (88–121)	0.843
Creatinine (mg/dl)	0.89 ± 0.35	0.9 ± 0.46	0.970
AST (U/l)	21 (17–26)	21 (16–27)	0.981
ALT (U/l)	16 (12–22)	19 (13–28)	0.155
Total bilirubin (mg/dl)	0.57 ± 0.30	0.51 ± 0.31	0.249
Albumin (g/l)	3.99 ± 0.69	4.10 ± 0.45	0.110
Sodium (mmol/l)	138 (135–140)	139 (137–140)	0.062
Potassium (mmol/l)	4.35 ± 0.51	4.29 ± 0.43	0.475
LDH (U/l)	245.9 ± 97.2	240.8 ± 76.4	0.706
Troponin I	0.22 ± 0.14	0.19 ± 0.53	0.879
Hemoglobin (mg/dl)	13.81 ± 4.23	14.11 ± 1.96	0.388
Platelets (10^3^/μl)	260 (161–339)	235 (191–295)	0.633
WBC (10^3^/μl)	10.87 (6.6–16)	8.37 (6–11.4)	**0.018**
Neutrophil (10^3^/μl)	7.5 (3.6–11.5)	5.1 (3.5–8.1)	0.061
Lymphocyte (10^3^/μl)	1.93 ± 1.1	1.9 ± 0.9	0.845
CRP (mg/l)	94.4 (51–148)	20.65 (6.5–50.4)	**<0.001**
NLR	4.58 (2.1–11.9)	2.93 (1.7–5.2)	**0.010**
PLR	183 (90–282)	129.2 (101–185)	**0.034**
CAR	25.51 (12–42)	5.04 (1.4–13)	**<0.001**
LVEF (%)	63 (60–65)	65 (58–65)	0.718
**Course of hospitalization and outcome**			
Hospital stay (d)	11 (9–12)	8 (7–10)	**<0.001**
ICU admission (n)	11 (27%)	56 (8%)	**<0.001**
In-hospital mortality (n)	5 (12%)	22 (3%)	**0.002**
**Imaging finding**			
Bilateral ground glass	26 (63%)	412 (55%)	0.331
Diffuse infiltration	13 (31%)	145 (19%)	0.060
**Severity of illness**			
Mild	2 (4.8%)	67 (9%)	0.360
Severe	39 (95.1%)	674 (90.9%)	

Bold letters have a p-value less than 0.05 and are statistically significant.

ALT: Alanine aminotransferase; AST: Aspartate aminotransferase; CAR: CRP/albumin ratio; CRP: C-reactive protein; ICU: Intensive care unit; L: Lymphocyte; LDH: Lactate dehydrogenase; LVEF: Left ventricular ejection fraction; N: Neutrophil; NLR: N/L ratio; NOAF: New onset atrial fibrillation; P: Platelet; PLR: P/L ratio; WBC: White blood cell.

When we look at the lung imaging findings, diffuse lung infiltraBold letters have a p-value less than 0.05 and are statistically significant and bilateral ground glass frequency were also similar in both groups (31 vs 19%, p = 0.060; 63 vs 55%, p = 0.331; Table 2).

Length of hospital and ICU stay were significantly longer in patients, who developed NOAF (p < 0.001, for both). In addition, in-hospital mortality was significantly higher in patients who developed NOAF (p = 0.002; Table 2).

The role of several risk factors in the development of NOAF was also evaluated via multivariate analysis. Multivariate logistic regression analysis was performed with values such as age, HT, HF, CRP, WBC, NLR, PLR and CAR, which were shown to be associated with AF formation during the hospital stay in univariate analysis. Multivariate logistic regression analysis demonstrated that CAR (odds ratio = 2.879; 95% CI: 1.063–7.793; p = 0.037) in blood samples taken at the time of admission to hospital and HF (odds ratio = 2.859; 95% CI: 1.313–6.230; p = 0.001) maintained independent importance in predicting NOAF in hospitalized SARS-CoV-2 patients (Table 3).

**Table 3.** Univariate and multivariate predictors of new onset atrial fibrillation in hospitalized patients with COVID-19.

Characteristics	Univariate analysis			Multivariate analysis		
	Odds ratio	95% CI	p-value	Odds ratio	95% CI	p-value
Age (years)	1.025	1.003–1.048	0.026			
Hypertension	1.968	1.045–3.708	0.036			
Heart failure	3.467	1.707–7.067	0.001	2.859	1.313–6.230	**0.008**
CRP	1.013	1.009–1.017	<0.001			
WBC	1.063	1.006–1.123	0.030			
CAR	1.632	1.416–1.882	<0.001	2.985	1.089–8.183	**0.037**
NLR	1.063	1.027–1.101	0.001			
PLR	1.002	1.000–1.003	0.021			

Bold letters have a p-value less than 0.05 and are statistically significant.

CAR: CRP/albumin ratio; CRP: C-reactive protein; L: Lymphocyte; N: Neutrophil; NLR: N/L ratio; P: Platelet; PLR: P/L ratio; WBC: White blood cell.

**Figure 2.**
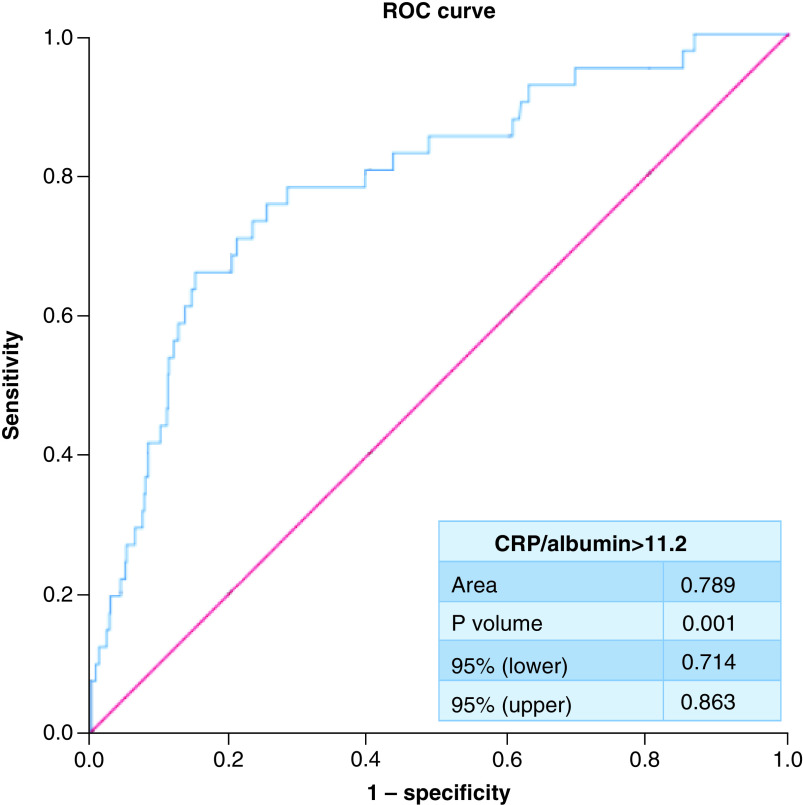
Receiver operating characteristic curve analysis for cut-off value of C-reactive protein/albumin ratio for detecting new onset atrial fibrillation. ROC: Receiver operating characteristic.

ROC curve analysis demonstrated that at a cutoff of ≥11.2 CAR in blood samples taken at the time of admission to hospital displayed 78% sensitivity and 71.8% specificity for detecting NOAF (area under ROC curve = 0.789; 95% CI: 0.714–0.863; p < 0.001) (Figure 2).

On the discharge documents of the patients, it was seen that 31 patients with NOAF returned to sinus rhythm, while five did not. These patients were treated according to the AF treatment guideline of the European Society of Cardiology. According to the European Society of Cardiology guidelines, patients with a CHA_2_DS_2_VASc score of 2 and above were given anticoagulant therapy [12]. Five patients in the NOAF group died in the hospital.

## Discussion

In our group of hospitalized patients with SARS-CoV-2 infection, NOAF was observed in 5.2%. Patients who developed NOAF during hospitalization for SARS-CoV-2 infection were more likely to have HT, HF and older age, compared with those who did not develop NOAF. The CRP level, WBC count, NLR, PLR and CAR in the blood samples taken at the time of admission to the hospital in patients, who developed NOAF, were significantly higher than those who did not develop NOAF during hospital stay. Among all these variables, CAR value was the most effective predictor for the development of AF in hospitalized SARS-CoV-2 patients.

AF is the most common arrhythmia in the population, which causes increased cardiovascular mortality and morbidity [12]. Moreover, one of the most common rhythm disorders clinicians encounter when looking at hospitalized patients is AF. It can be seen especially in the course of diseases that affect the respiratory tract, such as pneumonia. Several studies have shown an increased risk of NOAF in patients hospitalized for pneumonia. In a recent prospective study conducted by Pieralli *et al.*, the authors reported that 10.3% of the study population, who were admitted for community-acquired pneumonia (CAP), experienced NOAF during hospitalization. Cangemi *et al.* found a 9.5% incidence of NOAF formation in patients hospitalized for CAP [13,14]. SARS-CoV-2 is novel coronavirus infection, which predominantly affects the lungs and causes pneumonia. However, although the relationship between inflammation, pneumonia and AF is well known, data on the prevalence and predictors of NOAF, which may worsen the clinical course of SARS-CoV-2 infection, are lacking. To the best of our knowledge, this study is the first to evaluate the prevalence and predictors of NOAF and associated risk factors in hospitalized SARS-CoV-2 patients.

The prevalence of NOAF in our study population was found to be 5.2%. Although this rate is significantly higher than the prevalence values reported for the general population, it was significantly lower than the rates found in patients hospitalized for CAP. The reason for this can be explained by the fact that the patients in our study group had less comorbid diseases and their pneumonia severity was lower than the aforementioned studies. In addition, the fact that the patients in the mentioned studies were also selected from patients with more critical and intensive care needs may have increased the incidence of AF. In addition, we excluded patients with previous history of AF in our study. In the mentioned studies, prior AF history may have also increased the incidence of NOAF.

Although the pathophysiology of SARS-CoV-2 infection-related cardiac events, including arrhythmias, is not fully understood. SARS-CoV-2 infection can affect the cardiovascular system by multiple mechanisms, causing cardiac arrhythmias. Since acute cardiac damage, shock and arrhythmia have been seen in substantial numbers in the course of the disease, some authors have suggested the term ‘Acute COVID-19 Cardiovascular Syndrome’ [15]. SARS-CoV-2 infection can cause direct myocardial cell injury, myocardial oxygen supply/demand mismatch, hypoxia, enhanced systemic inflammation and catecholamine surge, increased thrombosis, and oxidative stress imbalance, which may all be related to the occurrence of AF [16,17].

Extensive data reveals that an inflammatory state and circulating TNF-α, IL-6 and IL-1β have been shown to increase in patients with SARS-CoV-2 infection [18]. It is now known that inflammation plays an important role in AF formation apart from traditional risk factors [4–6]. This relationship was explained by the infiltration of the atrium with inflammatory cells, myocyte necrosis and fibrosis formation. Previously, especially, inflammatory mediators such as CRP, IL-6 and TNF-α secreted during the inflammatory process were shown to induce AF development [4–6]. In addition, some studies have shown that serum CRP level increase is associated with increased AF development risk, frequent recurrence rate after catheter ablation and the need for more electrical cardioversion for AF [19–22].

Decreased levels of albumin, a negative acute-phase reactant, are associated with an increase in many cardiovascular diseases, including AF, independent of traditional risk factors [23]. Several studies have demonstrated that CAR, a new parameter of inflammation introduced in recent years, is superior to CRP and albumin levels alone in determining the inflammatory condition in cardiovascular diseases [24,25]. Additionally, a very recent study showed that the increase in CAR is independently associated with the development of NOAF [11].

In our study, CRP levels were significantly higher in SARS-CoV-2 patients, who developed AF, compared with those who did not. Although albumin levels were lower, it was not statistically significant. CAR was significantly higher in patients with AF. Accordingly, it can be said that the increasing systemic inflammatory activity in these patients is more common. In our multivariate analysis, we showed that CAR level in the blood, taken at the time of admission to the hospital, is the only strong and independent predictor of NOAF development in SARS-CoV-2 patients.

WBC level and its subtypes such as NLR and PLR have been shown as indicators of inflammation in various cardiovascular diseases. NLR is an inexpensive and easy-to-obtain systemic inflammatory marker that can be used in risk stratification in various cardiovascular diseases, in addition to the traditionally used inflammatory markers, especially in recent years. In addition, some studies have shown that it is a prognostic indicator of adverse cardiovascular events. Recently, several studies have shown that the increase in NLR is a predictor of AF development [7–9]. PLR, like NLR, is another inflammatory marker that has been studied in various cardiovascular patient groups in recent years and has proven to be of prognostic importance [26,27]. The PLR increase has also been shown to be associated with adverse cardiovascular events [26]. In another recent study, it was shown that the increase in PLR is an independent predictor of paroxysmal AF [10]. In our study, we found that patients with SARS-CoV-2 infection, who developed AF, had higher WBC, NLR and PLR levels at presentation, compared with those without AF. Inflammatory markers such as CAR, NLR and PLR were higher in patients with AF, making us reconsider the probability that the severity of the infection/inflammation may be a trigger for AF.

It is now a known fact that classical risk factors such as advanced age, HT, diabetes mellitus, presence of valvular heart disease and history of HF increase the risk of NOAF, as shown in many previous studies [12]. Indeed, in our study, patients who developed NOAF were older than those who did not. We also found that SARS-CoV-2 patients with comorbidities such as HF had a higher risk of developing NOAF, which has been shown with previous studies.

## Conclusion

SARS-CoV-2 infection may have triggered NOAF by causing an increase in various inflammatory markers that are proven to play an important role in the pathophysiology of AF. Among these inflammatory markers, we found that the ratio of two acute-phase reactants such as CRP and albumin, combined as a single index, was the most important independent predictor of NOAF development.

## Study limitations

This study has some limitations. The lack of Holter monitoring or long-term ECG monitoring for all patients is the main drawback of this study, and it is likely that silent AF may have been undetected. Hence, we may have underestimated the real incidence of AF in SARS-CoV-2 infection. Detailed echocardiography data were not available in all patients due to the high risk of viral transmission. Another limiting factor is the evaluation of CAR levels with only at admission of the hospital. We did not evaluate follow-up period, only a single measurement of CAR was used. There were a relatively small number of patients and the study was a single-center study. Another limiting factor is these results may be due to pneumonia secondary to SARS-CoV-2 or may be secondary to the systemic inflammatory response that can be seen in the course of SARS-CoV-2 infection. We cannot speculate that our result is specific for SARS-CoV-2 infection. Finally, because the drug treatment algorithm recommended by the Ministry of Health was applied to all hospitalized subjects and almost all subjects received the same agents for medical treatment, the specific role of drugs could not be compared in patients with and without NOAF. Larger and multicenter studies should be performed to better analyze all the possible predictors of AF. Nevertheless, we believe that our findings add valuable information to the current knowledge on the prevalence of NOAF and associated risk factors for the development of NOAF in SARS-CoV-2 patients.

**Summary points**

- COVID-19 is caused by SARS-CoV-2 and is a new RNA virus that primarily presents with acute pneumonia and severe respiratory distress syndrome.
- Although the pathophysiology of SARS-CoV-2 related cardiac events, including arrhythmias, is not fully understood, SARS-CoV-2 can affect the cardiovascular system by multiple mechanisms, causing cardiac arrhythmias such as atrial fibrillation (AF).
- The accumulated evidence has shown that besides the increase in C-reactive protein (CRP) level, the increase in new inflammatory markers such as the neutrophil/lymphocyte (L) ratio, platelet/L ratio, and CRP/albumin ratio (CAR), are also associated with the development of AF.
- The purpose of this study was to investigate new onset AF (NOAF) predictors in patients hospitalized for SARS-CoV-2, who developed NOAF on follow-up.
- A total of 782 patients were included in the study. NOAF was identified in 41 patients (5.2%).
- Patients who developed AF were older and had higher frequencies of hypertension and heart failure compared with patients without NOAF.
- Patients who developed NOAF during hospitalization had higher CRP level, white blood cell count, neutrophil/L ratio, platelet/L ratio and CAR levels compared with those who did not develop NOAF.
- Multivariate logistic regression analysis demonstrated that CAR in blood samples taken at the time of admission to hospital and heart failure maintained independent importance in predicting NOAF in hospitalized SARS-CoV-2 patients.
- Receiver operating characteristic analysis demonstrated that at a cutoff of ≥11.2 CAR in blood samples taken at the time of admission to hospital displayed 78% sensitivity and 71.8% specificity for detecting NOAF.
- A high level of CAR in blood samples is associated with an increased risk of developing NOAF in SARS-CoV-2.
